# Ischemic heart disease and stroke in male couriers: a cohort study using the national health insurance data and national employment insurance data

**DOI:** 10.3389/fpubh.2024.1416327

**Published:** 2024-07-11

**Authors:** Jiyoung Yoon, Jeehee Min, Eun Mi Kim, Jaiyong Kim, Inah Kim

**Affiliations:** ^1^Korea Employment Information Service (KEIS), Chungcheongbuk-do, Umsung-gun, Republic of Korea; ^2^Hanyang University Medical Center, Seoul, Republic of Korea; ^3^National Health Insurance Service (NHIS), Gangwon-do, Wonju, Republic of Korea; ^4^Hanyang University College of Medicine, Seoul, Republic of Korea

**Keywords:** male couriers, office workers, ischemic heart disease, stroke, cohort study

## Abstract

**Objectives:**

This study aimed to determine the risk of ischemic heart disease (IHD) and stroke among male couriers in Korea by linking the data from the National Health Insurance (NHI) and National Employee Insurance (NEI) databases.

**Methods:**

As of 2015, the NHI and NEI databases were linked using individual IDs. A cohort of male couriers, aged between 20 and 64 years, (*N* = 5,012) was constructed using the Korean Employment Insurance Occupational Classification (KECO-2007). For comparison, a cohort of male total wage workers (*N* = 5,429,176) and a cohort of office workers (*N* = 632,848) within the same age group were also constructed. The follow-up was conducted until 31 December 2020 to confirm the occurrence of IHD and stroke. The diagnoses were defined using the 10th revision of the International Statistical Classification of Diseases and Related Health Problems (ICD-10) codes. The criteria included medical services for more than 1 day of hospitalization or more than 2 outpatient visits. The age-standardized incidence ratio (SIR) was calculated to evaluate the risk of occurrence. The hazard ratio (HR) was calculated using the Cox model after adjusting for age, alcohol consumption, smoking, obesity, income level, and employment duration as confounding variables.

**Results:**

The SIR of IHD for couriers was 1.54 (95% CI 1.31–1.78), while for office workers, it was 1.08 (95% CI 1.06–1.10), compared to male total wage workers. The SIR for stroke was higher for couriers at 1.84 (95% CI 1.40–2.28) and lower for office workers at 0.86, compared to male total wage workers. Couriers had a higher SIR for stroke at 1.84 (95% CI 1.40–2.28) and lower for office workers at 0.86 (0.83–0.89). Compared to total wage workers, couriers had a significantly higher adjusted HR for IHD at 1.60 (95% CI 1.37–1.87) and a higher HR for stroke at 1.39 (95% CI 1.07–1.79). Compared to office workers, couriers had a significantly higher HR for IHD at 1.34 (95% CI 1.13–1.59) as well as for for stroke at 1.43 (95% CI 1.08–1.88).

**Conclusion:**

The incidence of IHD and stroke was higher among male couriers compared to male office workers and total wage workers, highlighting the need for implementing public health interventions to prevent IHD and stroke among couriers.

## 1 Introduction

A courier is a person or a company responsible for transporting packages or important documents (1, 2). According to the 2021 Local Area Labor Force Survey published by the National Statistical Office, the number of couriers as of October 2020 was 428,000, representing an increase of 9.7% from the previous year (3). Despite the rapidly growing number of couriers, their working conditions remain poor. Globally, the average number of working hours for couriers per day is more than 14 h, and they perform sustained high-intensity work, such as delivering for 6 days a week due to a large volume of orders (4, 5). The increase in deaths among couriers due to ischemic heart disease (IHD) or stroke has raised social awareness about the urgent need for implementing various policies to prevent such incidents (4, 6, 7).

IHD and stroke, which are among the leading causes of death worldwide, have been reported to be strongly associated with work (8–10). As of 2019, it has been reported that approximately 17.9 million workers die annually from cardio-cerebrovascular disease, accounting for 32% of occupational deaths worldwide (8). This finding can be attributed to occupational factors, such as long working long hours and night shifts, which increase the risk of developing IHD. Additionally, previous studies have revealed increased risks associated with some occupations that require intense physical labor or long working hours (9, 10). Specifically, couriering is among the occupations that require high physical labor intensity and long working hours.

This study aimed to determine the risks of IHD and stroke among male couriers in Korea by linking the data from National Health Insurance (NHI) and National Employee Insurance (NEI) databases.

## 2 Materials and methods

### 2.1 Data source

Two representative nationwide datasets were linked. First, since 2002, the NHI database has provided extensive data from 46,605,433 individuals out of a target population of 47,851,928. These data include information on health insurance type (employee insured, self-employed insured, and medical-aid beneficiary), health insurance status, medical treatment, and health examination managed by the National Health Insurance Service (NHIS) in Korea (11). This study used data from 2015 to 2020 regarding NHI eligibility, medical treatment records, and health examination results. Second, the NEI database provides data regarding the employment insurance enrollment history of workers nationwide, supplied by the Korea Employment Information Service (12). The data include approximately 15 million individuals' annual personal characteristics (age and sex), employment insurance enrollment status, occupation classification, and general information on the company, including business size, address, and standard industrial classification. This study included male couriers, office workers, and total wage workers using the NEI's insured occupation and business industry classification. The health data of these populations were merged with the NHI data using personally identifiable information. Additionally, the period during which each group maintained employment was confirmed using the data on the duration of health insurance type within the NHIS.

### 2.2 Study participants

**Figure 1:**
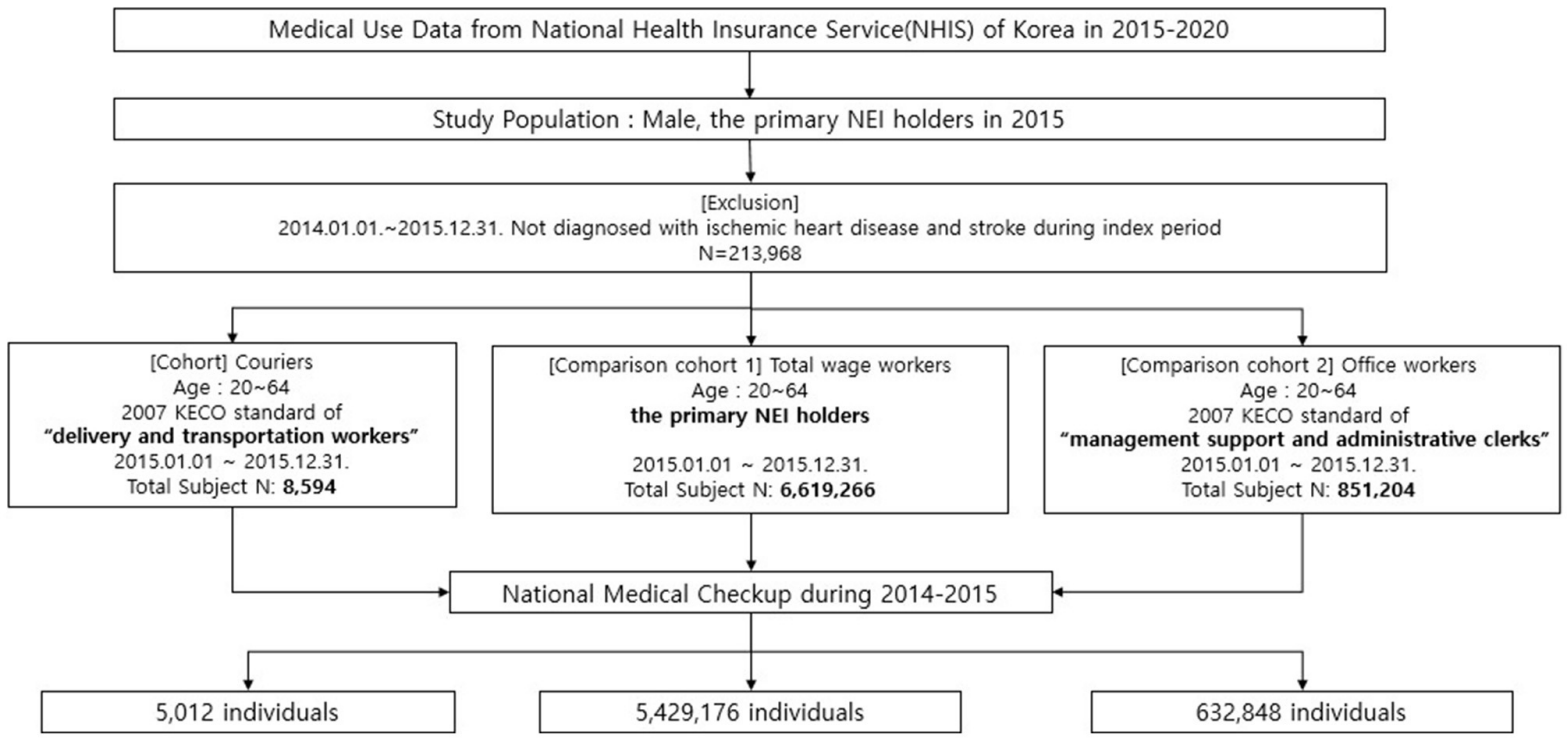
Population selection process.

Excluding individuals who were diagnosed with IHD or stroke in 2014–2015, the remaining participants were divided into three groups: the cohort group (couriers), the first comparison group (total wage workers), and the second comparison group (office workers) (Figure 1).

The cohort group (couriers) was restricted to male individuals because the proportion of female individuals among all couriers was approximately 1.5%. Male couriers aged 20–64 years were selected based on the standard Korean Employment Insurance Occupational Classification (KECO-2007) of “delivery and transportation workers.” Workers associated with businesses in the sectors of “courier services,” “transportation,” and “logistics” were included based on their workplace code. This approach led to the inclusion of small business owners and managers. Their classification was confirmed through the distribution based on types of health insurance subscription.

Two groups were selected as comparison groups. The comparison cohort 1 (total wage workers) comprised individuals whose insurance type was “employee-insured.” The comparison cohort 2 (office workers) comprised people aged 20–64 years with an occupational classification of “management support and administrative clerks.” Individuals with complete information on confounders were included in the final analysis. In all groups, male participants who underwent health examinations in 2014–2015 were included to adjust for age, alcohol consumption, smoking, obesity, income level, and employment duration, which are major risk factors for IHD and stroke. Finally, 5,012 couriers, 632,848 office workers, and 5,429,176 total wage workers were included in this study.

### 2.3 Variables

IHD and stroke were classified based on the findings of previous studies (11–13). This classification was based on the International Classification of Diseases, 10th Revision (ICD−10) codes for IHD (I20–I25) and stroke (I60–I64) diagnoses (13). Participants with a history of IHD (I20–I25) and stroke (I60–I64) between 1 January 2014 and 31 December 2015 were excluded. New cases were defined as occurrences between 1 January 2016 and 31 December 2020. To identify new cases of IHD and stroke, patients who used medical services for more than 1 day of hospitalization or had more than 2 outpatient records were included (13, 14).

The variables were defined as follows: (1) Person-years: Diagnosis of IHD (I20–I25) and stroke (I60–I64) during the follow-up period from 2015 to 2020 was considered a disease occurrence. The follow-up was terminated when disease occurred or the patient died; (2) income: This variable divided all subjects (100%) into 20 categories of 5% each, according to health insurance claim costs. The income variable was divided into four categories in the study (0%−25%, 25%−50%, 50%−75%, and 75%−100%); (3) employment duration: Employment status was defined based on the period during which the “employee insured” health insurance type was maintained. Loss to follow-up during the observation period indicated the end of the health insurance maintenance period; (4) obesity: This variable was defined as having a body mass index (BMI) of ≥25 kg/m^2^; and (5) alcohol consumption: This variable was classified as “yes” if alcohol consumption was > 40 g for those who drank once a week; and (6) smoking: This variable was classified as “yes” if the participants were current smokers.

### 2.4 Statistical analysis

Categorical variables are presented as frequencies and percentages, whereas continuous variables (e.g., age) are presented as means and standard deviations. The chi-squared test was used for categorical variables, and the F test was used for continuous variables to compare baseline characteristics. The crude incidence rate (per 100,000 individuals), age-dependent standardized incidence ratio (SIR), and 95% confidence interval (95% CI) were calculated. Event incidences were compared, and 95% confidence intervals (95% CI) were calculated using the Poisson distribution. Finally, the risk ratios for IHD and stroke by occupation were measured using the Cox proportional hazards model and Kaplan–Meier curves. Hazard ratios (HR) were measured after adjusting for age, employment duration, income level, obesity, alcohol consumption, and smoking status.

Analyses were performed using SAS Enterprise Guide software version 7.1 (SAS Institute, Inc., Cary, NC, USA). The chi-squared test, F-test, SIR, and Cox proportional hazards models were performed to calculate statistical significance with *P* values.

## 3 Results

### 3.1 Demographic characteristics of study participants

The study participants included 5,012 couriers, 632,848 office workers, and 5,429,176 total wage workers (Table 1). Couriers had a lower income level and a relatively shorter employment duration than office workers. Compared with office workers, couriers had a higher smoking rate (54.5%), a lower prevalence of obesity (42.1%), and a higher rate of alcohol consumption (50.9%). The same findings were observed for total-wage workers. There was no statistical difference between the groups in terms of age and employment duration. Regarding the income level, approximately 69.2% of the courier group belonged to the Q1 and Q2 quartiles, while 52.9% of the office workers belonged to the Q4 quartile. The obesity prevalence and alcohol consumption rates were higher in office workers, while the smoking rate was higher in the courier group.

**Table 1:** Demographic characteristics of the study participants.

**Group**		**Total wage workers (*****N*** **=** **5,429,176)**		**Couriers(*****N*** **=** **5012)**		**Office workers (*****N*** **=** **632,848)**		***p* ^b^**
		**N** ^a^ **(M** ^a^ **)**	**%**^a^ **(SD**^a^**)**	**N** ^a^ **(M** ^a^ **)**	**%**^a^ **(SD**^a^**)**	**N**^a^ **(M**^a^**)**	**%**^a^ **(SD**^a^**)**	
Person-year (mean)		25,566,193.0		24,628.4		3,129,470.0		-
Age		(42.9)	(10.0)	(43.8)	(9.7)	(43.6)	(8.8)	0.9
Income level	Q1 (0%−25%)	568,881	10.5	1,140	22.7	17,356	2.7	< 0.001
	Q2 (25%−50%)	1,080,834	19.9	2,331	46.5	87,508	13.8	
	Q3 (50%−75%)	1,765,194	32.5	1,364	27.2	159,753	25.2	
	Q4 (75%−100%)	1,731,043	31.9	149	3.0	334,871	52.9	
Employment duration (years)		(4.1)	(1.6)	(3.6)	(1.9)	(4.2)	(1.5)	0.5
Obesity	Yes	2,316,788	42.7	2,90	42.1	283,161	44.7	< 0.001
	No	3,112,388	57.3	2,112	57.9	349,687	55.3	
Smoking	Yes	2,352,182	43.3	2,264	45.2	179,215	39.5	< 0.001
	No	3,076,204	56.7	2,748	54.8	273,979	60.5	
Alcohol consumption	Yes	2,840,328	52.3	2,430	51.5	255,502	56.4	< 0.001
	No	2,589,810	47.7	2,582	48.5	197,915	43.6	

^a^N, number; M; mean; %, percent; SD, standard deviation. ^b^The chi-squared test was used for continuous variables, and the F test was used for categorical variables.

### 3.2 Incidence of ischemic heart disease and stroke

The age-dependent SIR of IHD (I21–I25) for couriers was 1.54 (95% CI 1.31–1.78), and for office workers, it was 1.08 (95% CI 1.06–1.10) (Table 2). The SIR of stroke (I60–I64) for couriers was 1.84 (95% CI 1.40–2.28), indicating a higher incidence compare to office workers, whose SIR was 0.86 (95% CI 0.83–0.89). The SIR of hemorrhagic stroke for couriers was 1.62 (95% CI 1.13–2.12), which was higher compared to office workers, who had an SIR of 0.84 (95% CI 0.80–0.86). In addition, compared to the total wage worker group, the courier group showed a significantly higher incidence of IHD (0.5, 95% CI 0.3–0.6) and stroke (0.7, 95% CI 0.4–0.9).

**Table 2:** Incidence of ischemic heart disease and stroke.

**Disease** ^**a**^		**Number of cases(N^b^)**	**Crude incidence rate(persons/100,000)**	**Age-standardized incidence ratio^d^(95% CI^b^)**	**β^c, d^(95% CI^b^)**
Ischemic heart disease (I20–I25)	Couriers	163	659.0	1.54^**^ (1.31–1.78)	0.5^***^ (0.3–0.6)
	Office workers	13,805	404.0	1.08^***^ (1.06–1.10)	0.1^***^ (0.3–0.6)
	Total wage workers	103,377	440.2	Reference	Reference
Stroke (I60–I64)	Couriers	67	270.9	1.84^***^ (1.40–2.28)	0.7^***^ (0.4–0.9)
	Office workers	3,700	140.0	0.86^***^ (0.83–0.89)	-0.2^***^ (-0.2–−0.1)
	Total wage workers	35,810	118.0	Reference	Reference
Hemorrhagic stroke (I60–I62)	Couriers	17	68.7	1.98^*^ (1.04–2.93)	0.7^**^ (0.3–1.2)
	Office workers	945	33.1	0.89^***^ (0.83–0.94)	-0.1^*^ (-0.2–0.0)
	Total wage workers	8,467	30.1	Reference	Reference
Ischemic infarction (I63)	Couriers	41	165.8	1.62^**^ (1.13–2.12)	0.5^**^ (0.2–0.8)
	Office workers	2,438	96.6	0.84^***^ (0.80–0.86)	−0.2^***^ (−0.3–−0.2)
	Total wage workers	24,727	77.7	Reference	Reference

^a^To identify new cases of IHD and stroke using ICD-10 codes, patients who used medical services for more than 1 day of hospitalization or had more than 2 outpatient records were included. ^b^N, Number; CI, 95% confidence intervals. ^c^The event incidences were compared by using Poisson distribution (95% CI). ^d*^p < 0.05, ^**^p < 0.01, ^***^p < 0.001.

### 3.3 Risks of ischemic heart disease and stroke

**Figure 2:**
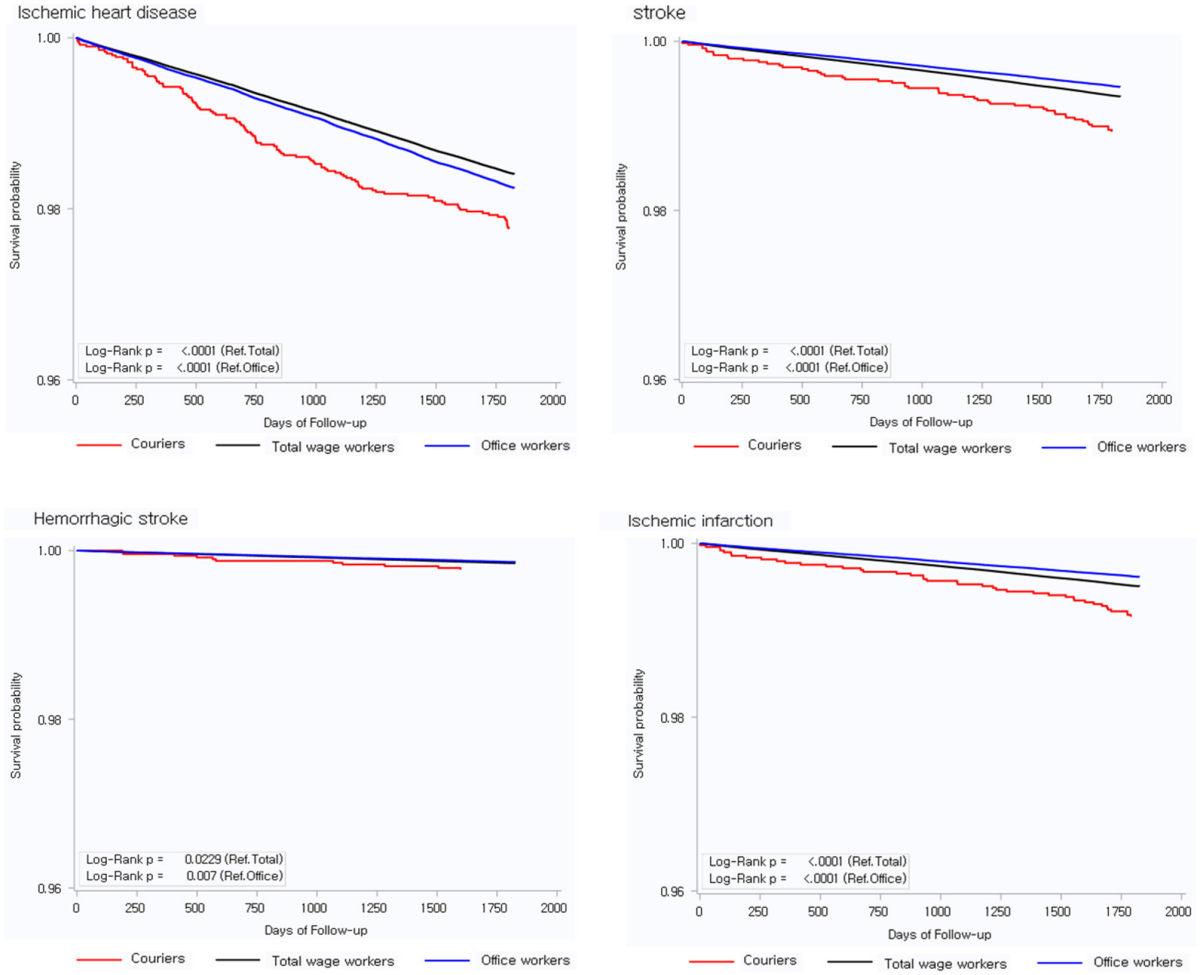
Kaplan-Meier curves.

Based on the Kaplan–Meier analysis, the incidence of IHD, stroke, hemorrhagic stroke, and ischemic infarction significantly increased among couriers over time (Figure 2). Similar results were obtained when analyzing the HRs of IHD and stroke according to occupation (Table 3). After adjusting for age, employment duration, income level, obesity, smoking, and alcohol consumption, compared with total wage workers, couriers had a significantly higher risk of IHD (HR, 1.60 [95% CI 1.37–1.87]) and stroke (HR, 1.39 [95% CI 1.07–1.79]). In addition, compared with office workers, couriers had a significantly higher risk of IHD (HR, 1.34 [95% CI 1.13–1.59]) and stroke (HR, 1.43 [95% CI 1.08–1.88]).

**Table 3:** Hazard ratios of ischemic heart disease and stroke in couriers.

**Disease**	**Ref** ^**a**^ **. Total wage workers** ^**b**^		**Ref. Office worker**	
	**cHR^a^ (95% CI^a, d^)**	**aHR^a, c^ (95% CI)**	**cHR (95%CI)**	**aHR (95%CI)**
Ischemic heart disease (I20–I25)	1.65^***^ (1.41–1.92)	1.60^***^ (1.37–1.87)	1.25^***^ (1.06–1.48)	1.34^***^ (1.13–1.59)
Stroke (I60–I64)	1.94^***^ (1.52–2.46)	1.39^*^ (1.07–1.79)	1.91^***^ (1.47–2.49)	1.43^*^ (1.08–1.88)
Hemorrhagic stroke (I60–I62)	2.08^**^ (1.30–3.35)	1.21 (0.69–2.14)	1.71 (0.99–2.44)	1.04 (0.56–1.95)
Ischemic infarction (I63)	1.72^***^ (1.26–2.33)	1.35^***^ (0.99–1.85)	2.09^***^ (1.54–2.85)	1.63^***^ (1.19–2.25)

^a^Ref: reference group, cHR: crude hazard ratio, aHR: adjusted hazard ratio, 95% CI: 95% confidence intervals. ^b^Excluding courier workers from total wage workers. ^c^Adjusted for age (1 year), changes in health insurance qualifications, income level, alcohol consumption, smoking, and obesity. ^d*^p < 0.05, ^**^p < 0.01, ^***^p < 0.001.

## 4 Discussion

Our study revealed that couriers had a higher risk of both IHD and stroke than total wage workers (IHD HR 1.60, 95% CI 1.37–1.87; stroke HR 1.39, 95% CI 1.07–1.79). The risks of IHD and stroke were also higher for couriers compared to office workers (IHD HR 1.34 [95% CI 1.13–1.59]; stroke HR 1.43 [95% CI 1.08–1.88]).

The couriers exhibited a higher smoking rate, which is a known risk factor for cardiovascular disease, and were slightly older. However, low rates of obesity and alcohol consumption, as well as low income levels, may influence the risk of cardiovascular disease. Therefore, HR was calculated by dividing the number of office workers as a reference and the number of total wage workers as a reference. Compared to the couriers, the office workers were older, had higher rates of obesity and alcohol consumption, and had higher income levels. The total-wage workers were comparatively younger and had lower obesity rates than the couriers. Despite these factors, couriers showed higher risks of IHD and stroke when compared to both reference groups.

Known occupational risk factors associated with IHD and stroke include long working hours, night shifts, or shiftwork (10, 15–18), high physical workload (19–21), and exposure to loud noises (22). A previous case–control study conducted in Japan showed an increased risk of IHD among motor vehicle drivers and cargo workers (23). This study suggests that the risk of IHD is high for occupations with high levels of physical labor. Additionally, it was revealed that excessively heavy lifting tasks are associated with a high incidence of IHD. This association also suggests that the occurrence of IHD may increase in situations with a similar working environment to that of courier workers (19). A previous meta-analysis study on stroke mentioned long working hours, shift work, and working in high and cold temperatures as occupational risk factors (24).

Furthermore, in Korea, a 2021 report indicated that couriers worked 11.5 h per day, exceeding the recommended “maximum acceptable work time (MAWT)” of 8 h (1). Couriers are exposed to long working hours, night shifts, and a high physical workload. In a previous study in Korea, which examined the risks of IHD and stroke among bus drivers who worked long hours (11–18 h), the incidence of IHD and stroke was higher in the crude model than in general workers (21). After adjusting for confounding variables, the association was not statistically significant. Nevertheless, in the present study, self-employed couriers were excluded, considering the coverage of the NEI data. Therefore, the risk can be even greater for those who do not fall under legal labor standards, such as self-employed couriers.

In Korea, social support, including special health examinations, has been provided since 2023 to prevent health problems caused by the courier work environment. This government health promotion program for workers who are at high risk of cerebrovascular and cardiovascular diseases includes in-depth screening for these conditions. The number of participants in this program increased to 20,000 in 2024. However, given that the target occupation group includes security guards, taxi and bus drivers, and courier workers, the screening coverage of the program remains insufficient considering the total population of courier workers. In addition, it has recently become possible for daily workers to sign up for employment insurance that will provide compensation in case of industrial accidents; however, the effectiveness of this new program requires follow-up analyses. In the United States, the Federal Motor Carrier Safety Administration was established to manage the vulnerable situation of these workers through policies, including measures such as monitoring the blood pressure of commercial vehicle drivers and offering medical care (25). A health policy approach of this nature is necessary.

The present study established two comparative cohorts to minimize the influence of healthy worker bias. Therefore, the total wage worker group included individuals engaged in heavy work, potentially increasing the risks of IHD and stroke, manual workers exposed to chemical hazards, and individuals in managerial positions characterized by high stress levels, long working hours, or nightshift work with no fixed schedules. To account for less exposure to physical and chemical risk factors for IHD and stroke, a cohort of office workers was also established. The healthy worker survival effect was considered by adjusting for the duration of employment. Given that the HR was consistently high for both groups in the full model, with all variables controlled, we concluded that couriers had higher risks of IHD and stroke.

This study had some limitations. First, disease occurrence may have been misclassified to some extent in this study, as the identification of disease occurrence was based on ICD-10 codes and the use of medical services rather than medical records or doctors' diagnoses. To minimize the misclassification, participants with ICD-10 codes who were hospitalized for 1 day or those with 2 outpatient records were defined as patients, in line with previous studies (11, 14). Moreover, since the misclassification in the NHI data is non-differential, the HRs presented in the results may be underestimated, considering that the bias effect tends to be nullified.

Second, alcohol consumption and smoking status were self-reported, which may have reduced the accuracy of the results. In addition, although the education level between the groups was expected to be different, it could not be controlled because there was no education level variable in the data. Further research is needed to explore this aspect.

Third, other confounding variables were not measured in this study. Previous studies proposed the E-value to verify whether an unmeasured confounder was corrected (26, 27). We measured the potential effect of strong bias due to residual confounding through E-value analysis to counteract the study's limitations. The E-value verifies the average/minimum value of the effect of unmeasured confounding variables on the exposure results. The standard average E-value is ≥1.7, with a minimum value of ≥1.2, indicating a low possibility of unmeasured confounding variables affecting exposure results. The average E-value calculated in this study was 1.86 with a minimum value of 1.31 for IHD and 2.45 with a minimum value of 1.57 for stroke.

One strength of this study is that it targeted the Korean population by linking the NHI and NEI databases. The NEI database, which includes data on self-employed workers and total employers, is the country's most comprehensive insurance database, covering 97.1% of the entire economically active population (28, 29). Furthermore, the NHI database from a single national insurer represents the use of medical services across the entire nation. The data used in this study included 7,000,000 male workers aged 15–64 years out of 15,760,000 employed individuals, as of 2015. Given that worker health examinations are conducted annually or biennially, this study included almost all wage workers and benefited from using nationally representative data, strengthening the validity of its findings.

## 5 Conclusion

To the best of our knowledge, this study is the first to confirm the risks of IHD and stroke in couriers using nationally representative linked data from the NEI and NHI databases. The results of this study further highlight the need for public health policies aimed at preventing IHD and stroke among couriers. Furthermore, additional well-designed studies should be conducted to identify potential occupational risk factors such as long working hours, night-shift work, and heavy lifting.

## Data availability statement

The data analyzed in this study is subject to the following licenses/restrictions: the data set was provided and analyzed on the NHIS system. The data is only accessible through the NHIS system. Requests to access these datasets should be directed to https://nhiss.nhis.or.kr/.

## Ethics statement

The studies involving humans were approved by the Local Ethics Review Board (Hanyang University Institutional Review Board, HYUIRB-202306-003). The studies were conducted in accordance with the local legislation and institutional requirements. Written informed consent for participation was not required from the participants or the participants' legal guardians/next of kin in accordance with the national legislation and institutional requirements.

## Author contributions

JY: Writing – review & editing, Writing – original draft, Visualization, Methodology, Formal analysis. JM: Writing – review & editing. EK: Writing – review & editing, Data curation, Visualization. JK: Writing – review & editing, Data curation. IK: Writing – review & editing, Supervision, Project administration, Methodology, Conceptualization.

## References

[1] Jang TW. A Study on the proper working hours of courier. In: Occupational Safety and Health Research Institute. (2021).

[2] Oxford Learner's Dictionaries. Oxford University Press (2023). Available online at: https://www.oxfordlearnersdictionaries.com (accessed October 3, 2023).

[3] National Statistical Office. Regional Employment Survey in the Second Half of 2021- Characteristics of Employed Persons by Industry and Region. National Statistical Office Press Release (2020). Available online at: http://kostat.go.kr (accessed October 3, 2023).

[4] Yonhap News Agency. 40 PCT of Delivery Drivers' Work Over 14 Hours Per Day During Peak Season: Survey (2020). Available online at: https://en.yna.co.kr/view/AEN20201201009100315 (accessed October 9, 2023).

[5] The Guardian. 14-hour Days and No Bathroom Breaks: Amazon's Overworked Delivery Drivers (2021) Available online at: https://www.theguardian.com/technology/2021/mar/11/amazon-delivery-drivers-bathroom-breaks-unions (accessed October 9, 2023).

[6] BBC NEWS. I Thought Maybe I Would Die. S Korea's Delivery Drivers Demand Change (2020). Available online at: https://www.bbc.com/news/world-asia-54775719 (accessed October 9, 2023).

[7] People. UPS Driver, 24, Dead From Suspected Heat Stroke After Passing Out in His Truck During Delivery (2022). Available online at: https://people.com/human-interest/ups-driver-dead-of-suspected-heat-stroke-after-passing-out-during-delivery (accessed October 9, 2023).

[8] World Health Organization. WHO/ILO Joint Estimates of the Work-Related Burden of Disease and Injury (2021). Available online at: https://www.who.int/publications/i/item/9789240034945 (accessed October 9, 2023).

[9] Lee S Lee H Kim HS Koh SB. Incidence, risk factors, and prediction of myocardial infarction and stroke in farmers: a Korean nationwide population-based study. J Prev Med Public Health. (2020) 53:313–22. 10.3961/jpmph.20.156 33070503 PMC7569019

[10] Yook JH Lee DW Kim MS Hong YC. Cardiovascular disease risk differences between bus company employees and general workers according to the Korean National Health Insurance Data. Ann Occup Environ Med. (2018) 30:32. 10.1186/s40557-018-0242-z 29760932 PMC5941761

[11] Lee J Lee JS Park SH Shin SA Kim KW. Cohort profile: the National Health Insurance Service–national sample cohort (NHIS-NSC), South Korea. Int J Epidemiol. (2017) 46:e15. 10.1093/ije/dyv319 26822938

[12] KEIS homepage. (2024). Available online at: https://eng.keis.or.kr/eng/subIndex/2234.do (accessed January 9, 2024).

[13] Choi HG Koh YS Lee SW. Increased risk of coronary heart disease with hysterectomy in young women: a longitudinal follow-up study using a national health screening cohort. Maturitas. (2022) 157:49–56. 10.1016/j.maturitas.2021.10.009 35120672

[14] Choe HJ Park S Han KD Moon MK Koo BK. Contribution of hypertriglyceridemia to ischemic cardiovascular disease in Korean women: a nationwide population-based study. J Clin Lipidol. (2022) 16:83–93. 10.1016/j.jacl.2021.11.008 34896034

[15] Lin RT Chien LC Kawachi I. Nonlinear associations between working hours and overwork-related cerebrovascular and cardiovascular diseases (CCVD). Sci Rep. (2018) 8:9694. 10.1038/s41598-018-28141-2 29946079 PMC6018699

[16] Kang MY Park H Seo JC Kim D Lim YH Lim S. Long working hours and cardiovascular disease: a meta-analysis of epidemiologic studies. J Occup Environ Med. (2012) 54:532–7. 10.1097/JOM.0b013e31824fe192 22576460

[17] Kim I Koo MJ Lee HE Won YL Song J. Overwork-related disorders and recent improvement of national policy in South Korea. J Occup Health. (2019) 61:288–96. 10.1002/1348-9585.12060 31025505 PMC6620743

[18] Li J Pega F Ujita Y Brisson C Clays E Descatha A. The effect of exposure to long working hours on ischaemic heart disease: a systematic review and meta-analysis from the WHO/ILO joint estimates of the work-related burden of disease and injury. Environ Int. (2020) 142:105739. 10.1016/j.envint.2020.105739 32505014 PMC7339147

[19] Petersen CB Eriksen L Tolstrup JS Søgaard K Grønbaek M Holtermann A. Occupational heavy lifting and risk of ischemic heart disease and all-cause mortality. BMC Public Health. (2012) 12:1070. 10.1186/1471-2458-12-1070 23231790 PMC3538157

[20] Holtermann A Mortensen OS Burr H Søgaard K Gyntelberg F Suadicani P. The interplay between physical activity at work and during leisure time–risk of ischemic heart disease and all-cause mortality in middle-aged Caucasian men. Scand J Work Environ Health. (2009) 35:466–74. 10.5271/sjweh.1357 19851700

[21] Raum E Rothenbacher D Ziegler H Brenner H. Heavy physical activity: risk or protective factor for cardiovascular disease? A life course perspective. Ann Epidemiol. (2007) 17:417–24. 10.1016/j.annepidem.2006.12.008 17395479

[22] Hwang WJ Hong O. Work-related cardiovascular disease risk factors using a socioecological approach: implications for practice and research. Eur J Cardiovasc Nurs. (2012) 11:114–26. 10.1177/1474515111430890 22357786

[23] Fukai K Furuya Y Nakazawa S Kojimahara N Hoshi K Toyota A. A case control study of occupation and cardiovascular disease risk in Japanese men and women. Sci Rep. (2021) 11:23983. 10.1038/s41598-021-03410-9 34907236 PMC8671491

[24] Yang M Yoo H Kim SY Kwon O Nam MW Pan KH. Occupational risk factors for stroke: comprehensive review. JoS. (2023) 25:327–37. 10.5853/jos.2023.01011 37813670 PMC10574301

[25] Federal Motor Carrier Safety Administration. What Is the Effect on Driver Certification Based on FMCSA Hypertension Stages? (2014) Available online at: https://www.fmcsa.dot.gov/faq/what-effect-driver-certification-based-fmcsa-hypertension-stages (accessed January 11, 2024).

[26] VanderWeele TJ Ding P. Sensitivity analysis in observational research: introducing the E-value. Ann Intern Med. (2017) 167:268–74. 10.7326/M16-2607 28693043

[27] Sjölander A Greenland S. Are E-values too optimistic or too pessimistic? Both and neither! *Int J Epidemiol*. (2022) 51:355–63. 10.1093/ije/dyac018 35229872 PMC9082795

[28] Kim ST Seong SC. National Health Insurance Statistical [Yearbook]. National Health Insurance Service. (2016).

[29] Jun KY Park J Oh KW Kim EM Bae JS Kim I. Epidemiology of ALS in Korea using nationwide big data. J Neurol Neurosurg Psychiatry. (2019) 90:395–403. 10.1136/jnnp-2018-318974 30409890 PMC6581156

